# Cognitive screening in the emergency department: Agreement between the patient‐ and caregiver‐completed AD8

**DOI:** 10.1002/alz.087105

**Published:** 2025-01-03

**Authors:** James Galske, Anna Sather, Tonya Chera, Ula Hwang, Christopher R Carpenter, Cameron Gettel

**Affiliations:** ^1^ University of Connecticut School of Medicine, Farmington, CT USA; ^2^ ICahn School of Medicine at Mount Sinai, New York, NY USA; ^3^ Yale School of Medicine, New Haven, CT USA; ^4^ NYU Langone Health, New York, NY USA; ^5^ Mayo Clinic, Rochester, MN USA

## Abstract

**Background:**

Impaired cognition is prevalent in older adults in the emergency department (ED), but many cases go unrecognized. The Ascertain Dementia 8 (AD8) is a validated, self‐administered screening tool that can be used by patients and caregivers to assess for early cognitive changes associated with dementia. However, little is known regarding the degree of alignment between respondents in the ED setting. Our objective was to assess the agreement between the patient‐(pAD8) versus caregiver‐completed AD8 (cAD8).

**Methods:**

We conducted a prospective observational study of community‐dwelling older adults aged 65+ years seeking emergency care and their caregivers between October 2021 and November 2023. Patients with a diagnosis of dementia indicated in the electronic health record were excluded. Collected by trained research assistants, AD8 scores ranged from 0‐8, with scores 2+ indicating cognitive impairment. Patients and caregivers were blinded to the others’ AD8 rating. The intraclass correlation coefficient (ICC) was used to assess the agreement of the presence or absence of cognitive impairment based on pAD8 and cAD8 scores.

**Results:**

Our analytic sample included 538 dyads, of which 34.7% of patients were male with a mean age of 73.5. A total of 132 (24.5%) patients scored themselves as cognitively impaired versus 201 (37.4%) patients were scored as cognitively impaired by their caregivers. Between groups, an ICC of 0.519 (95% CI 0.454‐0.578) demonstrated moderate agreement in identifying cognitive impairment based on pAD8 and cAD8 scores.

**Conclusion:**

Approximately 1 in 4 older adults without a dementia diagnosis in the emergency department identified themselves as having cognitive impairment, with caregivers even more likely to identify cognitive impairment in accompanied older adult patients. With current guidance suggesting that the pAD8 be used in the absence of the cAD8, these findings highlight important differences to account for based on the respondent when screening for cognitive impairment.

